# The Culture Of Health In Early Care And Education: Workers’ Wages, Health, And Job Characteristics

**DOI:** 10.1377/hlthaff.2018.05493

**Published:** 2019-05

**Authors:** Jennifer J. Otten, Victoria A. Bradford, Bert Stover, Heather D. Hill, Cynthia Osborne, Katherine Getts, Noah Seixas

**Affiliations:** School of Public Health, University of Washington, in Seattle.; School of Public Health, University of Washington.; School of Public Health, University of Washington.; Evans School of Public Policy and Governance, University of Washington.; Lyndon B. Johnson School of Public Affairs, University of Texas at Austin.; School of Public Health, University of Washington.; School of Public Health, University of Washington

## Abstract

Little is known about the health of the 2.2 million early care and education (ECE) workers responsible for the care, well-being, and success of the approximately ten million children younger than age six enrolled in ECE, or the extent to which ECE environments and employers play a role in workers’ health. The purpose of this analysis was to describe the health of an ECE worker sample by wage and by job and center characteristics and to begin to explore the relationships between these factors and workers’ health. Our data indicate that ECE workers earn low wages and experience poor mental well-being and high rates of food insecurity. Lower-wage workers worked at centers with more children enrolled in subsidy programs and were more likely to work at centers that did not offer health insurance, paid sick leave, or parental or family leave. Policies and programs that raised workers’ wages or mandated the provision of meals to both children and workers could better support teacher health and the quality of ECE for children. Our results suggest that the culture of health in ECE settings and equity-related outcomes could be improved by helping centers provide support and flexibility to teachers (for example, offsetting workers’ benefit costs or reducing teacher-to-child ratios to reduce stress) who are managing their own health in the context of demanding work.

A high-quality early care and education (ECE) workforce is central to the care, well-being, and success of the approximately ten million children younger than age six who are enrolled in ECE and are at their most critical stage of growth and development.^1,2^ Since the 1970s ECE professionals, scholars, and policy makers have recognized that workforce quality is critical to caregiving quality.^2^ Accordingly, the focus of research and of federal and state initiatives has been on bolstering the quality of care, in large part through improving the education/training and job satisfaction of workers and reducing staff turnover.^1,2^ However, little attention has been given to the health of the ECE workforce, how workers’ health affects caregiving quality, whether ECE environments and employer supports play a role in this, and the extent to which local and state initiatives could be helpful. This gap in knowledge could be significant, given the role that ECE workers likely play in shaping the culture of health in ECE settings—that is, the environments, habits, and relationships related to physical and emotional health that are essential to young children’s lifelong learning, health, and behavior.

The current 2.2 million paid ECE workers represent a highly vulnerable workforce.^1,3^ Women and people of low educational and socioeconomic status are overrepresented in this workforce.^1,4^ In 2017 ECE workers earned a median wage of $10.72 per hour or $22,290 per year, with 86 percent of ECE center–based educators working with infants and toddlers and 67 percent of those working with preschoolers (children ages 3–5) making less than $15 an hour.^1,5^ In 2017 ECE workers earned less than two-thirds of the median for all occupations in all states.^1^ These poverty-level wages are insufficient for ECE workers to meet their basic needs, and 43–54 percent of the workers are enrolled in public assistance programs (such as Medicaid)—a rate substantially higher than the 21 percent for elementary and middle school teachers.^1,4^ The material deprivation and stress associated with low income are known to have effects on people’s health, including higher rates of chronic disease and shorter life expectancies.^6–8^ Recent studies suggest that 25–30 percent of ECE workers do not have health insurance.^1,3,9^ In addition to low wages, ECE working conditions are characterized by long hours, high turnover, and physically and emotionally demanding jobs, and there is some evidence to suggest that these conditions are related to adverse effects on mental well-being.^10,11^

A handful of studies that examined the health of ECE teachers consistently found that they had higher rates of overweight, obesity, and chronic disease (hypertension, diabetes, asthma, and migraines), compared to national averages or to women with similar demographic characteristics.^3,9,12–14^ In some cases, these studies also found that ECE teachers have poorer mental health, higher levels of stress, and higher prevalence of several risky behaviors (such as smoking, lack of physical activity, and unhealthy diet) than population norms. Two studies found that ECE workers report clinically depressive symptoms at rates approximately two to five times greater than national averages, and another study found that the workers often feel emotionally strained and distressed, which is related in part to their work.^3,11,15^ However, very few studies have examined the possible relationships between job and worksite characteristics and workers’ health or health behaviors.

More attention is needed to increase understanding of how the structure of ECE jobs might affect workers’ health (that is, physical and mental well-being) and to consider health-supportive policy and program opportunities. The purpose of this analysis was to describe the health of a sample of ECE workers by wage and by job and center characteristics to begin to explore the relationships between these factors and workers’ health. Dimensions of ECE jobs and centers that could influence the health and well-being of ECE workers include compensation (pay, benefits, and leave), classroom structure (number of students and teacher-to-child ratios), center environment (aspects of workplace culture such as whether the center is for profit or nonprofit and what the minimum education requirements are for teachers), and centers’ participation in subsidy and quality improvement programs. We hypothesized that lower-wage ECE workers would have poorer self-reported health and food security and would be more likely to be employed at centers that did not offer health insurance or paid sick or family leave. This study contributes to the literatures on the ECE workforce and ECE quality by exploring how worker and job characteristics, work conditions, and child care policies may influence the culture of health in ECE.^16^

## Study Data And Methods

### STUDY DESIGN

The current analysis used baseline data from an ongoing study titled Exploring the Effects of Wage on the Culture of Health in Early Childhood Education Centers, which examines how wages and wage changes are affecting ECE workers’ health and ECE care environments in Washington State and Texas. The study is collecting four waves of data in a prospective cohort over three years, taking advantage of wage changes resulting from recent city and state minimum wage laws: The hourly minimum wage will be $15 in Seattle as of January 1,2019, for Schedule 2 employers and $13.50 in Washington State as of January 1, 2020, for all employers; there is no similar change in Austin, Texas, where the federal hourly minimum wage of $7.25 remains in effect. The study was designed to shed light on the relationships between wages and wage changes, workers’ health, and the provision of high-quality and healthful care. The Institutional Review Board at the University of Washington approved all protocols.

### SAMPLE, RECRUITMENT, AND DATA COLLECTION

In the period August–December 2017, we enrolled forty-nine ECE centers (sixteen in Seattle, sixteen in South King County, and seventeen in Austin) in the study. To be eligible for the study, centers had to serve children ages 0–6 and have no plans to close in the following two years. After centers were enrolled, study staff members visited each center to meet with directors and recruit workers. During the site visits, directors were given a center-focused questionnaire. The questionnaire asked about the wages, practices, and characteristics of their center, and measures relevant to this analysis are described below.

At the in-person worker recruitment meetings, study staff members explained the study and the consent process and collected contact information for workers interested in participating. Both full-time and part-time employees were eligible. All 504 workers who expressed interest were emailed a link to an online survey or mailed a paper copy of baseline surveys, according to their preference. The survey included several validated tools with questions about workers’ wages and jobs, mental and physical well-being, food security, chronic diseases, and health behaviors. Specific survey measures are described below. In the period September 2017–January 2018, 366 workers completed baseline measures—144 (76 percent) in Seattle, 98 (65 percent) in South King County, and 124 (76 percent) in Austin.

To help provide further context to baseline findings, the study team conducted six ninety-minute focus groups in July 2018, one with directors and another with teachers at each of the three study sites. Directors were asked about the health of their staff, how workers’ health affects their ability to care for children, and what their center does to support the health of its staff. Teachers were asked about aspects of their work that help or hinder their ability to take care of their own health. Both groups were asked to reflect on specific findings about workers’ health from the baseline surveys. Additional details on the sample, recruitment, and data collection are in online appendix exhibit A1.^17^

### MEASURES

The worker survey collected information from teachers about job characteristics, work conditions, demographic characteristics, and self-reported health. In a separate survey, center directors provided information on center staffing structure, compensation, and participation in state subsidy and quality improvement programs. The variables used in this analysis are described below and in appendix exhibit A1.^17^

### ► MEDIAN WAGE CATEGORY:

To examine differences between workers in higher- and lower-wage positions, we created a binary variable for worker hourly wage at the sample median for each of the three study sites and combined them into two median wage categories: hourly wage less than the site median and hourly wage greater than or equal to the site median. The sample hourly medians were $17.35 in Seattle, $14.08 in South King County, and $14.82 in Austin.

### ► WORKERS’ HEALTH:

The 12-Item Short Form Health Survey (SF-12) was used to assess both physical and mental well-being.^18^ The twenty-item Center for Epidemiologic Studies Depression Scale—Revised was used to measure depressive symptoms.^19^ Stress was measured using the fourteen-item Perceived Stress Scale.^20^ Food security was measured using the validated six-item U.S. Household Food Security Survey Module.^21^ Questions that ask whether participants had ever been told by a doctor that they had high blood pressure, high cholesterol, or diabetes were sourced from the National Health Interview Survey.^22^ Body mass index was calculated from self-reported height and weight. To measure physical activity, participants completed the long version of the International Physical Activity Questionnaire,^23^ which categorizes people into groups that have low, moderate, or high levels of activity. Diet was assessed with the thirty-item Dietary Screener Questionnaire.^24^ Additionally, participants responded to standard questions about sleep and smoking behaviors.^22,25^

### ► CENTER CHARACTERISTICS:

A set of variables collected in the center director survey was appended to each worker’s data according to the center at which they were employed. These included the number of staff members and children; average hourly wage; monthly enrollment fee for four-year-olds; profit status; National Association for the Education of Young Children accreditation; participation in the Quality Rating and Improvement System; participation in the Child and Adult Care Food Program, which provides reimbursements for meals that meet healthy criteria; receipt of state or city subsidies for care; provision of health insurance, paid sick leave, or parental or family leave to employees; and minimum education requirements for teachers.

### ANALYSIS

We calculated descriptive summary statistics for workers, their self-reported health, and their center or job characteristics by median wage category. We tested differences in health and center or job characteristics by median wage category, using chi-square tests for categorical variables and *t*-tests for continuous variables. Study team members conducted broad deductive coding of the focus-group transcripts based on the interview guide and wrote analytic memos summarizing the themes, key differences, and other notable findings using a common template.

### LIMITATIONS

This study had a few limitations. First, it was limited to three sites in two states and used nonprobability samples, which were not representative of all ECE centers or workers in the sites studied. Second, self-reporting of health conditions may have led to measurement error. Despite these limitations, this study contributes to greater understanding of the role of workplace health as a component of the culture of health in ECE by exploring the associations between wage, center and worker characteristics, and workers’ health.

## Study Results

The final sample included 366 ECE workers from forty-nine ECE centers. We present the characteristics of the centers in appendix exhibit A2.^17^ By design, the centers varied in terms of the number of children they served, the number of employees they had, and the average hourly wage they paid to full-time employees. Roughly half of the ECE centers were nonprofit, and the rest were for profit. Fifteen of them had minimum education requirements of more than a high school diploma or GED.

### WORKER AND JOB CHARACTERISTICS

Exhibit 1 presents worker demographics by median hourly wage categories. The majority of workers were female (94 percent) and non-Hispanic white (68 percent). Fifteen percent were black or African American, and 21 percent were of Hispanic ethnicity. The majority of workers were full time, defined as working at least thirty-five hours a week (85 percent), had a college degree (65 percent), and had an average of ten years of experience in ECE. Only 19 percent were members of a professional ECE society. Twenty-three percent reported using any public food assistance. Only 10 percent did not have health insurance (additional detail on insurance is presented in exhibit 2).

Workers with lower wages were younger, had less education, and had worked fewer years in the industry, compared to their colleagues with higher wages (exhibit 1). In addition, those with lower wages were more likely to be nonwhite or of Hispanic ethnicity and to be enrolled in public food assistance programs.

### SELF-REPORTED WORKER HEALTH

Self-reported worker health is shown in exhibits 3 and 4. Workers in our sample experienced poor mental well-being and high rates of food insecurity (that is, they lacked the ability to consistently access enough food for an active and healthy life), with more mixed results for physical well-being and health behaviors. Further delineation of these findings and comparisons of our sample to other populations are provided in the “Discussion” section below (additional details are in appendix exhibit A4).^17^

### WORKERS’ CENTER CHARACTERISTICS

The characteristics of the centers where our worker sample were employed are shown in exhibit 5 and analyzed in the “Discussion” section below. Overall, we found associations between lower wages and centers that served fewer children, had fewer employees, had lower student enrollment fees, served more subsidized families, and participated in the Child and Adult Care Food Program and the Quality Rating and Improvement System. In addition, lower wages were associated with centers that did not offer paid sick leave, health insurance, or parental or family leave.

### FOCUS-GROUP FINDINGS

Three key themes emerged from the focus groups that helped us contextualize our survey findings (see illustrative quotes in appendix exhibitA6).^17^ First, when asked about the health of ECE workers, participants from all sites emphasized the importance of emotional and mental health. Washington focus-group participants talked very little about physical health, other than the adjustment to germs that new teachers face. In contrast, Austin focus-group participants described both mental and physical health challenges of the work and how the two were intertwined. Everyone agreed that the work was emotionally demanding and stressful. Some felt that the workers attracted to the profession were often emotionally vulnerable. When teachers were asked what they did to promote their own health, they focused on stress management techniques when they were off work, such as taking naps, playing with pets, going on walks, and various other techniques to “mellow out and let everything go,” as one teacher put it.

Second, societal and parental disrespect were consistently described as a key source of stress for ECE workers. A phrase that was often repeated during the focus group was that “you better love your job,” which implies that there were few rewards—monetary or otherwise—for doing it. ECE workers viewed their work as undervalued in terms of compensation, status in society, and daily recognition from parents. The low status of their jobs seemed particularly unfair, given the growing scientific evidence on the importance of high-quality care early in life.

Third, all of the directors we spoke to wanted to support their teachers’ mental and physical health, but staffing and other resource constraints made that difficult. Specific constraints to providing flexibility and support included not having assistant teachers or floating staff members, not offering paid sick days, and not having the financial resources to choose to have class sizes below the maximum allowed by regulations. Teachers described a wide degree of variation in support for their health from centers and directors. Even when centers provided supports such as paid sick leave, workers often felt they could not take advantage of them due to staffing limitations. While parents were told to keep children home with symptoms of sickness (for example, a high fever), workers generally did not have the luxury of staying home.

## Discussion

This analysis examined early care and education workers’ health and center characteristics overall and as a function of wage. We added to the emerging but limited research on ECE workforce health. Our data indicate that ECE workers earn low wages and experience poor mental well-being and high food insecurity, with more mixed results for physical well-being and health behaviors. We found associations between lower wages and centers that served fewer children, had fewer employees, had lower student enrollment fees, served more subsidized families, and offered fewer employee benefits. Focus-group findings helped contextualize these results. Teachers and directors described the stress of caring for young children and depicted a workforce whose members felt undervalued by society. Directors felt constrained in the health supports they could offer workers while simultaneously offering affordable care for children. They felt that these factors, combined with low wages and the lack of flexibility on the job, contributed to poor worker mental well-being.

Mental well-being and food insecurity rates in our sample were much worse than population norms yet similar to other ECE samples. While depression rates are known to be higher in women and lower-income people, depression rates in our sample (40 percent fell into clinically significant categories of depression, data shown in online appendix exhibit A4)^17^ were double the prevalence of depression found in a nationally representative US sample of women with family incomes below 100 percent of the federal poverty level (40 percent versus 20 percent) and nearly quadruple the prevalence found in women overall (10.4 percent).^26^ This rate also exceeds the 26 percent prevalence of depressive symptoms found in low-wage nursing home employees, a group of low-wage workers whose members also experience emotionally and mentally stressful working conditions.^27^ Yet our finding is consistent with that found in an ECE worker population in North Carolina (36 percent).^3^ Consistent with these findings, the SF-12 mental health subscales in our population were all below average (exhibit 3). The average stress score in our sample (23.6) indicated slightly more stress than population norms (19.6).^28^ However, our sample had less stress than a comparable group of family home ECE providers: 60 percent of our respondents had moderate stress and 7 percent of them had high stress (data shown in online appendix exhibit A4),^17^ while 63 percent of the family home providers had high stress, as measured by the Perceived Stress Scale—10.^9^

The results of this study offer a look at the remarkably high food insecurity rates in ECE workers, especially in lower-wage workers. Food insecurity was an issue for 42 percent of our sample, compared with US food insecurity rates of 11.8 percent.^29^ A 2017 Arkansas workforce study of instructional ECE workers also recently found a 40 percent food insecurity rate overall, with a 50 percent rate in workers who cared primarily for younger children (that is, infants and toddlers).^30^ Moreover, very low food security—which indicates multiple disruptions to regular eating patterns, reduced food intake, and hunger—was experienced by 20 percent of our sample, as compared with 4.5 percent in the US population.^29^ Food insecurity is consistently negatively associated with health, including increased rates of mental health problems, depression, and chronic health conditions.^31^ In a sample of low-wage nursing home employees, 49 percent experienced food insufficiency sometimes and 67 percent experienced it often, and this was associated with depressive symptoms but not financial strain.^27^

The chronic disease rates in our sample were better than population norms and rates in recent ECE worker studies. While two such studies found rates of 55–65 percent for obesity and 22–24 percent for overweight, this study found that only 34 percent of workers were obese and 27 percent were overweight—rates that are also lower than the US population prevalence of 40 percent for obesity and 32 percent for over-weight.^3,9,32^ However, these studies used measured height and weight to calculate body mass index, while our values were based on self-reported data, which are known to underreport obesity. Similarly, the 17 percent in this study who reported that a doctor had diagnosed them as having high blood pressure was lower than the US population norm (29 percent) and a recent ECE worker sample (36 percent).^3,33^ Doctor-diagnosed diabetes was also lower than the US population norm (7.0 percent versus 9.4 percent).^34^ These findings raise the hypothesis that differences in physical well-being might be more associated with geographic region (in other words, “place matters”), while similarities in mental well-being findings suggest that social and emotional health might be more tied to the job for this workforce.

In terms of health behaviors and self-rated physical health, our findings were mixed. Our sample reported eating fruit and vegetables 2.3 times per day (exhibit 4), slightly lower than the 2.6 times reported in another ECE worker sample.^3^ Our sample reported much higher levels of aerobic activity than the US population norm and a recent ECE study (86 percent versus 53 percent and 28 percent meeting recommendations, respectively).^3,35^ However, our study used self-reported data, while the other ECE study used objective accelerometer data.^3^ ECE workers in our study averaged 6.5 hours of sleep a night, slightly less than national recommendations of 7–8 hours but similar to the results in two ECE worker studies.^3,9^ The SF-12 physical health subscales indicated that, on average, our sample scores were similar to population norms in terms of physical functioning but worse in terms of pain and general health.

Interesting trends emerged when we examined workers’ center characteristics overall and by wage. In particular, lower-wage workers worked at centers with lower tuition rates and with more low-income, subsidized families and that were less likely to offer health-related benefits. Other studies have found that wage increases are constrained by family tuition rates and other program costs, such as workers’ benefits and the number of enrolled subsidized families.^36,37^ In particular, ECE centers that raise family tuition rates to increase wages risk reducing enrollments of low-income families through either self-selection or center caps.^37^ This is because state- and city-level subsidies often do not fully cover tuition costs for low-income families, and centers or families are faced with having to offset deficits. Together, this suggests that there are equity issues at play that might affect health, for both workers and children. In the focus groups, directors described how they as employers could support healthier and more equitable environments through initiatives that partially paid workers’ wages, fully covered workers’ health benefits, or subsidized families.

### Policy Implications

The cross-sectional nature of these baseline data and our use of descriptive analyses prevent us from making causal conclusions. Nonetheless, our findings have several policy implications. Overall, wage level was positively associated with multiple dimensions of workers’ health. Policies that raised wages without creating other unintended consequences (for example, reduced enrollment of subsidized families) could better support workers’ health and the quality of early care and education for children. For example, in the period 2000–2003, Washington State funds were used to pay additional wages to ECE workers based on educational advancement.^38^ This resulted in significantly more positive interactions between children and teachers in the pilot sites, compared to those in the comparison sites.^38^ Raising wages might be particularly helpful in reducing teachers’ food insecurity, if those wages contributed to higher family income.^39^ A greater effort to mandate the provision of meals to both children and teachers would also be a possible approach to improving teachers’ food security and modeling healthy eating for children.

The high rates of depression, even among the higher-paid teachers, may require more targeted interventions. This finding supports current efforts to invest in mental health consultants to work with teachers, directors, and parents to develop strategies to help children who are struggling with behavioral problems. It is likely that interventions using such consultants may also improve the well-being of the workers by reducing stress and making their jobs easier, but this hypothesis would need to be tested.

More broadly, our results suggest that the culture of health in ECE settings and equity-related outcomes could be improved by helping centers provide support and flexibility to teachers managing their own health in the context of demanding work. These resources could include offsetting the cost of workers’ benefits, reducing teacher-to-child ratios to reduce stress, or subsidizing floater staff members. For centers receiving state or local subsidies, this investment could be partially accomplished by reimbursing full tuition for subsidized families.

## Supplementary Material

Appendix

## Figures and Tables

**EXHIBIT 1:** Demographic characteristics of 366 early care and education (ECE) workers, by hourly wage, 2017

		Workers whose hourly wage is:		
			
	All workers	Below the site median (*n* = 175)	At or above the site median (*n* = 179)	*p* value
Sex				0.401
Female	341	165	164	
Male	22	9	13	
Mean age (years)	37	33	41	<0.001
Race				0.008
White	229	93	130	
Black or African American	52	33	13	
Asian	29	15	16	
Other	28	15	12	
Ethnicity				0.061
Hispanic	76	42	28	
Non-Hispanic	282	131	145	
Education				<0.001
Less than high school	7	5	1	
High school graduate or GED	59	51	6	
Some college in ECE	60	30	29	
Associate or bachelor's degree	201	81	114	
Master’s degree or higher	27	3	22	
Marital status				<0.001
Never married	165	107	55	
Married	135	42	86	
Divorced, separated, widowed, or other	57	23	33	
Mean number in household	2.75	2.75	2.68	0.585
Mean household income ($)^a^	54,210	36,893	69,871	<0.001
Median hourly wage ($)	15	13	17.5	<0.001
Has health insurance^b^				0.001
Yes	331	150	171	
No	35	25	8	
Receives any food assistance^c^				<0.001
Yes	84	58	24	
No	282	117	155	
Mean years worked in ECE	10	6	14	<0.001
Member of a professional society^d^				0.003
Yes	64	18	41	
No	277	141	130	
Job title				<0.001
Center director	29	4	22	
Program coordinator	17	1	16	
Lead teacher	122	44	73	
Teacher	95	54	39	
Assistant teacher	78	63	13	
Other^e^	25	9	16	

**SOURCE** Authors’ analysis of data from the ECE worker survey from the study. **NOTES** Survey responses do not always sum to the full sample size because participants did not answer or chose to skip a survey response. Twelve records without wage data were not included in the wage columns. Chi-square tests were used for categorical variables, *t*-tests for continuous variables, and median regression for comparison of medians for computation of *p* values. Appendix exhibits A1 and A3 contain more detailed descriptions of the survey and percentages and standard deviations of the data in the table (see note 17 in text).

a Combined incomes of all members of the household ages fifteen and older (including the ECE worker) over the past twelve months. This includes money from jobs; net income from business, farm, or rent; pensions; dividends; interest; Social Security payments; and any other monetary income.

b Detailed findings on health insurance are in exhibit 2.

c Includes Supplemental Nutrition Assistance Program (*n* = 43); the Special Supplemental Nutrition Program for Women, Infants, and Children (WIC) (*n* = 27); farmers market WIC program (*n* = 6); food bank (*n* = 15); reduced-price or free school lunch (*n* = 32); and other (*n* = 1). People participating in more than one program were counted only once.

d Such as the National Association for the Education of Young Children, the National Association for Family Child Care, and the National Institute on Out-of-School Time.

e Something else, including two aides.

**EXHIBIT 2:** Health insurance status of 366 early care and education (ECE) workers, by hourly wage, 2017

		Workers whose hourly wage is:		
			
	All workers	Below the site median	At or above the site median	*p* value
Has health insurance				0.001
Yes	331	150	171	
No	35	25	8	
Insured through employer				
Yes	208	84	117	0.001
No	158	91	62	
Covered by spouse’s or partner’s insurance				
Yes	44	13	30	0.009
No	322	162	149	
Has Apple Health, Medicaid, or similar government-assisted insurance				0.006
Yes	39	27	11	
No	327	148	168	
Has Medicare				—^a^
Yes	4	2	2	
No	362	173	177	
Covered by the Department of Veterans Affairs				
Yes	0	0	0	—^a^
No	366	175	179	
Purchased insurance directly from the company				
Yes	17	13	4	0.026
No	349	162	175	
Covered by the Indian Health Service				—^a^
Yes	0	0	0	
No	366	175	179	
Has other insurance				0.068
Yes	16	11	4	
No	350	164	175	

**SOURCE** Authors’ analysis of data from the ECE worker survey from the study. **NOTES** Sample sizes are in exhibit 1. Survey responses do not always sum to the full sample size because participants did not answer or chose to skip a survey response. Twelve records without wage data are not included in the wage columns. Chi-square tests, *t*-tests, and median regression were used as explained in the notes to exhibit 1. Appendix exhibits A1 and A3 contain more detailed descriptions of the survey and percentages and standard deviations of the data in the table (see note 17 in text). Apple Health is the name for Medicaid in Washington State.

a Not applicable because there were too few respondents in some cells to reliably compute statistics.

**EXHIBIT 3:** Mental and physical well-being of 366 early care and education (ECE) workers, by hourly wage, 2017

		Workers whose hourly wage is:		
			
	All workers	Below the site median	At or above the site median	*p* value
**MENTAL WELL-BEING**				
Mean depression CESD-R score^a^	15.79	16.78	14.97	0.125
Categorical depression based on CESD-R score:^a^				
Major, probable, or possible depression	26	18	8	0.084
Subthreshold depression^b^	120	57	58	
No clinically significant depression	213	95	111	
Mean perceived stress score’	23.6	24.7	22.8	0.022
Categorical stress based on perceived stress score:^c^				
Low stress	118	46	68	0.082
Moderate stress	217	110	99	
High stress	25	14	11	
Mean SF-12^d^ Mental Health Subscale scores				
Vitality	48.69	48.96	48.43	0.594
Social functioning	44.66	42.95	46.19	0.007
Mental health	46.56	46.25	46.74	0.670
**PHYSICAL WELL-BEING**				
Workers told by a doctor they had:				
High blood pressure				
Yes	62	26	35	0.240
No	304	149	144	
High cholesterol				
Yes	54	19	34	0.032
No	312	156	145	
Diabetes				
Yes	25	9	15	0.230
No	341	166	164	
Mean BMI^e^	29.08	28.74	30.06	0.042
Categorical BMI:^e^				
Underweight	6	4	2	0.754
Normal	133	65	65	
Overweight	96	45	46	
Obese	119	53	62	
Mean SF-12^d^				
Physical Health Subscale scores				
Physical functioning	50.27	50.37	50.23	0.890
Bodily pain	46.94	47.19	47.19	0.999
General health	43.96	42.51	45.15	0.034

**SOURCE** Authors’ analysis of data from the ECE worker survey from the study. **NOTES** Sample sizes are in exhibit 1. Survey responses do not always sum to the full sample size because participants did not answer or chose to skip a survey response. Twelve records without wage data are not included in the wage columns. Chi-square and *t*-tests were used as explained in the notes to exhibit 1. Appendix exhibits A1 and A4 contain more detailed descriptions of measures and percentages and standard deviations of the data in the table (see note 17 in text).

a The Center for Epidemiologic Studies Depression Scale—Revised (CESD-R) depression score is determined by responses to twenty questions and ranges from 0 to 60, with lower scores indicating fewer depressive symptoms.

b See the appendix for an explanation of “subthreshold.”

c The Perceived Stress Scale (PSS)—14 score is determined by responses to fourteen questions and ranges from 0 to 56 (low stress: <20; moderate stress: 20–36; high stress: >36).

d The 12-Item Short Form Health Survey (SF-12) consists of twelve items. Subscales range from 0 to 100, with higher scores representing above-average health status, and are designed to have a mean score of 50 with a standard deviation of 10 in a representative sample of the US population.

e “Underweight” is defined as having a body mass index (BMI) of <18:5 kg=m^2^. “Normal” weight, “overweight,” and “obese” are defined as having BMIs of 18.5–24.9, 25.0–29.9, and 30.0 or more, respectively.

**EXHIBIT 4:** Food security and health behaviors of 366 early care and education (ECE) workers, by hourly wage, 2017

		Workers whose hourly wage is:		
	All workers	Below the site median	At or above the site median	*p* value
**FOOD SECURITY**				
Mean food security score^a^	1.83	2.23	1.44	0.001
Categorical food security score^a^				
High security or marginal security	212	86	119	0.002
Low security	75	39	34	
Very low security	71	45	23	
**HEALTH BEHAVIORS**				
Mean number of times per day ate:^b^				
Fruit and vegetables	2.33	2.13	2.57	0.002
Dairy products	0.92	0.92	0.94	0.776
Sweets	1.22	1.32	1.13	0.128
Mean MET minutes per week^c^	3,477	3,731	3,301	0.310
Categorical physical activity based on MET minutes per week^c^				
Low activity	51	22	25	0.294
Moderate activity	171	75	91	
High activity	138	73	62	
Mean hours usually slept per night	6.50	6.60	6.41	0.122
Categorical sleep per night:				
Slept 7 or more hours	180	90	85	0.419
Slept less than 7 hours	179	81	91	
Used tobacco in the past 30 days^d^				
Yes	47	24	23	0.826
No	316	150	154	
Smoked e-cigarettes in the past 30 days^d^				
Yes	20	11	9	0.599
No	345	163	170	

**SOURCE** Authors’ analysis of data from the ECE worker survey from the study. **NOTES** Sample sizes are in exhibit 1. Survey responses do not always sum to the full sample size because participants did not answer or chose to skip a survey response. Twelve records without wage data are not included in the wage columns. Chi-square and t-tests were used as explained in the notes to exhibit 1. Appendix exhibits A1 and A4 contain more detailed descriptions of measures and percentages and standard deviations of the data in the table (see note 17 in text).

a The score is determined using the six-item US Household Food Security Survey Module. Raw scores range from 0 to 6 (high security or marginal security: 0 or 1; low security: 2–4; very low security: 5 or 6).

b The Dietary Screener Questionnaire asks respondents, “During the past month, how often did you eat…” for a number of food items. We grouped responses into food groups and calculated the number of times per day foods from each group were eaten.

c The International Physical Activity Questionnaire (long version) asks twenty-seven questions about five activity domains. The instrument produces outcome measures in weekly energy expenditures by intensity (MET) minutes per week.

d We used National Health Interview Survey questions to ask participants how often they had smoked or used e-cigarettes in the past thirty days.

**EXHIBIT 5:** Characteristics of the centers of 366 early care and education (ECE) workers, by hourly wage, 2017

		Workers whose hourly wage is:		
			
	All workers	Below the site median	At or above the site median	*p* value
Number of children				0.028
50 or fewer	105	56	43	
51–75	104	58	44	
More than 75	138	56	78	
Number of employees				0.012
14 or fewer	101	52	43	
15–30	167	90	75	
More than 30	76	25	47	
Average hourly wage of full-time employees				
$7.00–$13.00	79	56	19	<0.001
$13.01–$15.00	127	58	65	
$15.01–$21.00	154	57	94	
Monthly enrollment fee for a 4-year-old				
$750 or less	44	28	11	0.003
$751–$1,250	167	82	80	
$1,251–$2,100	135	55	80	
Profit status				0.946
Nonprofit	165	77	83	
For profit	148	73	73	
Affiliated with community college or university	38	16	17	
Center participates in CACFP	155	93	52	<0.001
Center participates in state QRIS	286	144	131	0.040
Center accredited by NAEYC	91	41	48	0.463
Center accepts state-level subsidies^a^	309	153	144	0.074
Center has at least one child who is enrolled in a state-level subsidy program	258	134	112	0.017
Center has more than 25% of enrolled children in a state-level subsidy program	94	64	26	<0.001
Center accepts city-level subsidies’^b^	183	88	85	0.598
Center has at least one child enrolled in a city-level subsidy program	112	51	51	0.804
Center offers health insurance				<0.001
Yes	252	110	134	
No	101	65	32	
Center offers paid sick leave				<0.001
Yes	321	141	170	
No	45	34	9	
Center offers parental or family leave				<0.001
Yes	281	116	156	
No	60	50	7	
Among centers that offer parental or family leave:				0.100
Offers unpaid leave	198	88	104	
Offers paid leave	83	28	52	
Minimum education required for teachers				0.621
High school diploma or GED	278	132	139	
More than high school diploma or GED	88	43	40	

**SOURCE** Authors’ analysis of data from the center director questionnaire in the study survey. **NOTES** Sample sizes are in exhibit 1. Survey responses do not always sum to the full sample size because participants did not answer or chose to skip a survey response. Chi-square tests were used as explained in the notes to exhibit 1. Appendix exhibits A1 and A5 contain more detailed descriptions of the source survey and percentages and standard deviations for the data in the table (see note 17 in text). A fuller version of this exhibit appears in the appendix. CACFP is Child and Adult Care Food Program. QRIS is Quality Rating and Improvement System. NAEYC is National Association for the Education of Young Children.

a Includes the Washington State Department of Social and Health Services and the Texas Child Care Management Services subsidy programs for child care.

b Includes the Seattle Child Care Assistance Program and the Texas Workforce Solutions Capital Area Child Care Services.
