# Supplementary material for: The Culture Of Health In Early Care And Education: Workers’ Wages, Health, And Job Characteristics
Source: Health Aff (Millwood). Author manuscript; Available in PMC 2021 May 29. (PMC8164392; doi:10.1377/hlthaff.2018.05493)
Supplement: Appendix [file NIHMS1602563-supplement-Appendix.pdf]

Appendix Exhibit A1: Additional information on study sample, recruitment, data collection, and measures.

Sample, recruitment, and data collection

From August to December 2017, we enrolled 49 ECE centers and then 370 individuals who worked at those centers. Resource and referral agencies provided lists of licensed child care centers with contact information for Seattle, South King County (SKC), and Austin. After excluding Head Start and state pre-school programs, center directors in Seattle (n=165), SKC (n=86), and Austin (n=366) were contacted via email, mail, and phone to participate in the study. To be eligible for the study, centers had to serve children between the ages of 0 to 6 and have no plans to close in the next two years. Interested and eligible centers were asked to respond to an online screener with questions about their location and average wage paid to their employees. Ninety-eight centers completed the online screener [Seattle n=26 (16%), SKC=16 (19%), and Austin=56 (15%)].

Using the screener variables to maximize variation in center location and wage, research staff called interested center directors to review study details and obtain consent to reach the recruitment goal of 49 enrolled centers (Seattle=16, SKC=16, and Austin=17). After enrollment, study staff visited each center to meet with directors and to recruit workers. During the site-visit, directors were provided a center-focused questionnaire with a stamped return envelope. Directors received a $75 gift card after returning the questionnaire. The questionnaire asked about the wages, practices, and characteristics of their center and measures relevant to this analysis are described below.

At the in-person worker recruitment meeting, research staff explained the study and the consent process and collected contact information for those interested in participating. To be eligible to participate, participants had to be at least 18 years old, able to read and speak English, and work in a position where they care for children. Both full-time and part-time employees were eligible. All those who expressed interest (n=504) were e-mailed a link to an online survey or mailed a paper copy of baseline surveys, per their preference. The survey took approximately 45 minutes to complete and workers were given a $30 gift card after returning the survey. Study data were collected and managed using secure REDCap electronic data capture tools hosted at the University of Washington.(18) The survey included several validated tools with questions about their wages and job, mental and physical well-being, food security, chronic disease, and other health behaviors. Specific survey measures are described below. A total of 366 workers completed baseline measures [Seattle n=144 (76%); SKC=98 (65%), Austin=124 (76%)] from September 2017 to January 2018.

To help provide further context to baseline findings, the study team conducted six 90-minute focus groups, one with directors and one with teachers by site, in July 2018. The focus group participants were volunteers recruited via email from the sample of baseline participants who had completed the survey. Participants received an incentive payment of $25. Focus groups ranged in size from two to ten participants (total n=26) and were moderated by experienced facilitators using an interview guide. Directors were asked about the health of their staff, how worker health affects their ability to care for children, and what the centers do to support the health of their staff. Teachers were asked about aspects of their work that help or hinder their ability to take care of their own health. Both groups were asked to reflect on

specific findings about worker health from the baseline surveys. The focus groups were audio recorded and transcribed.

Measures

The worker survey collected information from teachers about job characteristics, work conditions, demographic characteristics, and health outcomes. On a separate survey, center directors provided information on center staffing structure and compensation, and participation in state subsidy and quality improvement programs. The variables used in this analysis are described below and in Table 2 footnotes.

Median Wage Category: To examine differences between workers in higher- and lower-wage positions, we created a binary variable for worker hourly wage at the sample median for each of the three study sites and combined them into two median wage categories: hourly wage less than site median, hourly wage greater than or equal to site median. Sample medians were $17.35/hr. in Seattle; $14.08/hr. in SKC; and $14.82/hr. in Austin.

Worker Health: The 12-item short form health survey (SF-12) assessed both physical and mental well-being. The SF-12 consists of 12 items and estimates health status subscale scores across eight health concepts. These concepts include physical functioning, role-physical, role-emotional and mental health using two questions each; bodily pain, general health, vitality, social functioning using one question each. Subscales range from 0-100, with higher scores representing above average health status, and are designed to have a mean score of 50 with a standard deviation of 10 in a representative sample of the U.S. population. Due to a survey typo, we were unable to score role-physical or role-emotional and thus are unable to calculate

the physical and mental health composite scores for baseline. Mental well-being subscales included are mental health, vitality, and social functioning. Physical well-being subscales included are physical functioning, bodily pain, and general health.

The 20-item Center for Epidemiologic Studies Depression Scale-Revised (CESD-R) was used to measure depressive symptoms. Example prompt statements include “I could not shake off the blues” or “I could not get going”. The CESD-R Depression Score is determined by 20 questions and ranges from 0-60, with lower scores being fewer depressive symptoms. Standard scoring protocols were followed where a total depressive symptom score (range 0-60) is created by summing item responses (scored 0-3 and reversed coded when appropriate) with scores ≥ 16 indicating risk of clinical depression (i.e., major or minor depression). Scores allow for categorization into major, probable, possible, and subthreshold depression (scores <16 are categorized as “no clinical significance”). Subthreshold are scores above 16 but do not have two or more Diagnostic and Statistical Manual, 5th edition symptom groups occurring either nearly every day for the past two weeks, or 5-7 days in the past week and are considered clinically relevant because the probability of clinically validated diagnosis is high.

Stress was measured using the 14-item Perceived Stress Scale intended to measure how unpredictable, uncontrollable, and overloaded individuals find their life circumstances. Participants respond to items experienced over the past month (e.g., “felt difficulties were piling up so high you could not overcome them”, “been able to control irritations in your life”) on a 5-point Likert scale (0=never, 4=very often). The PSS-14 scores range from 0 to 56, with higher scores indicating greater perceived stress. Approximation of stress levels are as follows:

less than 20 equals low stress; 20 to 36 equals moderate stress; and more than 36 equals high stress.

Food security was measured using the validated 6-item U.S. Household Food Security Survey Module and refers to the ability to access enough food for an active and healthy life. This tool asks participants about food eaten in their household over the past year and whether they were able to afford the food they needed. Responses of "often," "sometimes," "yes," "almost every month" and "some months but not every month" contribute to a raw score of 0 to 6, with high scores indicating very low food security.

U.S. National Health Interview Survey questions asked if participants had ever been told by a doctor that they had high blood pressure, high cholesterol, or diabetes.

Body mass index was calculated from self-reported height and weight as kilograms divided by meters squared and categorized into underweight, normal weight, overweight, and obese (<18.5, 18.5-24.9, 25.0-29.9, ≥30 kg/m^2, respectively).

To measure physical activity, participants completed the International Physical Activity Questionnaire (IPAQ) long version (i.e., 27 questions about 5 activity domains: job-related, transportation, housework, sedentary behavior, time spent sitting) which categorizes individuals into low, moderate or high levels of activity.The IPAQ produces outcome measures in minutes of weekly energy expenditures by intensity (MET-minutes/week) and can be used to categorize individuals into low, moderate or high levels of activity as follows: Low: Those individuals who do not meet criteria for Moderate or High. Moderate (meets U.S. physical activity recommendations): (a) 3 or more days of vigorous-intensity activity of at least 30 minutes per day, or (b) 5 or more days of moderate-intensity activity and/or walking of at least

30 minutes per day, or (c) 5 or more days of any combination of walking, moderate-intensity or vigorous intensity activities achieving a minimum total physical activity of at least 600 MET-minutes/week. High: (a) vigorous-intensity activity on at least 3 days achieving a minimum total physical activity of at least 1500 MET-minutes/week, or (b) 7 or more days of any combination of walking, moderate-intensity or vigorous-intensity activities achieving a minimum total physical activity of at least 3000 MET-minutes/week.

Diet was assessed with a 30-item Dietary Screener Questionnaire (DSQ) and scored to provide daily number of times eaten for fruit and vegetables, dairy/calcium, added sugars, whole grains/fiber, red meat, and processed meat. The DSQ asks "During the past month, how often did you eat…" for a number of food items. Responses are Never, 1 time last month, 2-3 times last month, 1 time per week, 2 times per week, 3-4 times per week, 5-6 times per week, 1 time per day, 2-3 times per day, 4-5 times per day, 6 or more times per day. We grouped questions into food groups and calculated the number of times per day foods from a group were eaten. We present fruit and vegetable (summed from six questions about fruits, salad, fried potatoes, potatoes, beans, and vegetables), dairy (summed from two questions about milk and cheese), and sweets (summed from seven questions on soda, juice, sweet drinks, candy, donuts, cookies, and ice cream) consumption.

Additionally, workers responded to standard questions about sleep and smoking behaviors.

<u>Center Characteristics:</u> A set of variables collected in the center director's survey were appended to each worker's data according to the center at which they were employed. These included the number of staff and children; average hourly wage; monthly enrollment fee for 4-

year-olds; profit status; National Association for the Education of Young Children accreditation; Quality Rating and Improvement System (QRIS) program participation; Child and Adult Care Food Program (CACFP) participation (i.e., provides reimbursements for meals that meet healthy criteria); receipt of state or city subsidies for care; provision of health insurance, paid sick leave, or parental/family leave to employees; and, minimum education requirements for teachers.

Appendix Exhibit A2: ECE Center Characteristics (n=49)

	N (%)	Median (Range)
**Number of children**		56 (18-167)
50 or less	20 (42.6)	
51-75	16 (34.0)	
More than 75	11 (23.4)	
**Number of employees**		16 (5-67)
14 or less	21 (44.7)	
15-30	22 (46.8)	
More than 30	4 (8.5)	
**Average hourly wage of full-time employees**		14.5 (8-20.8)
$13 or less	16 (34.0)	
$13.01-$15	13 (27.7)	
More than $15	18 (38.3)	
**Monthly enrollment fee for 4 yr olds**		1012 (485-2100)
$750 or less	9 (20.0)	
$751-$1250	22 (48.9)	
$1251-1750	12 (26.7)	
More than $1750	2 (4.4)	
**State**		
Washington	332 (65.3)	
Texas	17 (34.7)	
**Profit status**		
Non-profit	23 (48.9)	
For-profit	21 (44.7)	
Community college or university affiliated	3 (6.4)	
**CACFP[a] participation**	24 (53.3)	
**State QRIS[b] participation**	40 (81.6)	
**NAEYC[c] accredited**	12 (24.5)	
**Accepts state-level subsidy[d]**	43 (87.8)	
At least 1 child currently enrolled on state-level subsidy program	40 (81.6)	
More than 25% of enrolled children on state-level subsidy program	19 (38.8)	
**Located in cities with a city-level subsidy program**	36 (73.5)	
Accepts city-level subsidy[e]	24 (49.0)	
At least one child currently enrolled on city-level subsidy program	17 (34.7)	
**Pays for employee medical benefits**	28 (58.3)	
For full-time employees (30 or more hours per week)	27 (55.1)	
For part-time employees	6 (12.2)	
For salaried employees only	1 (2.0)	
**Offers paid sick leave**	39 (79.6)	
For full-time employees (30 or more hours per week)	35 (71.4)	
For part-time employees	17 (34.7)	
For salaried employees only or director only	2 (4.1)	
**Offers parental/family leave**		
No leave offered	13 (28.3)	
Offers unpaid leave	22 (47.8)	
For full-time employees	17 (34.7)	
For part-time employees	10 (20.4)	

Offers paid leave	11 (23.9)
For full-time employees (30 or more hours per week)	9 (18.3)
For part-time employees	3 (6.1)
For salaried employees only	1 (2.0)
**Minimum education requirement for teachers**	
High school diploma/GED	34 (69.4)
More than high school diploma/GED	15 (30.6)

SOURCE: Study Survey (Center Director Questionnaire)

[a]Child and Adult Care Food Program
[b]Quality Rating and Improvement System
[c]National Association for the Education of Young Children
[d]This includes the Washington State Department of Social and Health Services (DSHS) and the Texas Child Care Management Services (CCMS) state subsidy programs for child care
[e]City programs include City of Seattle Child Care Assistance Program and Texas Workforce Solutions Capital Area Child Care Services

Appendix Exhibit A3. ECE Worker (n=366) Demographics by Hourly Wage, further detail (n=366)

	All subjects	< site median hourly wage (n=175)	≥ site median hourly wage (n=179)	p-value
**Overall, N (%)**	366 (100)	175 (49)	179 (51)	
**Sex, N (%)**				.401
Female	341 (94)	165 (95)	164 (93)	
Male	22 (6)	9 (5)	13 (7)	
**Age, mean (SD)**	37 (13)	32.94 (12.06)	40.85 (12.70)	< .001
**Race, N (%)**				.008
White	229 (68)	93 (60)	130 (76)	
Black, African American	52 (15)	33 (21)	13 (8)	
Asian	29 (9)	15 (10)	16 (9)	
Other	28 (8)	15 (10)	12 (7)	
**Ethnicity, N (%)**				.061
Hispanic	76 (21)	42 (24)	28 (16)	
**Education, N (%)**				< .001
< High School	7 (2)	5 (3)	1 (1)	
High School grad/GED	59 (17)	51 (30)	6 (3)	
Some college in early education	60 (17)	30 (18)	29 (17)	
Associate or BA	201 (57)	81 (48)	114 (66)	
Masters or higher	27 (8)	3 (2)	22 (13)	
**Marital status, N (%)**				< .001
Never married	165 (46)	107 (62)	55 (32)	
Married	135 (38)	42 (24)	86 (49)	
Divorced, separated, widowed, other	57 (16)	23 (13)	33 (19)	

	All subjects	< site median hourly wage (n=175)	≥ site median hourly wage (n=179)	p-value
**Number in household, mean (SD)**	2.75 (1.27)	2.75 (1.28)	2.68 (1.24)	.585
**Household income (US$)[a], mean (SD)**	54,210 (41,637)	36,893 (27,707)	69,871 (44,856)	< .001
**Hourly wage (US$), mean (SD)**	15.64 (4.10)	13.03 (2.01)	18.20 (4.00)	< .001
**Hourly wage (US$), median**	15.00	13.00	17.50	<.001
**Health Insurance, N (%)**				.001
Currently has health insurance	331 (90)	150 (86)	171 (96)	
No current health insurance coverage	35 (10)	25 (14)	8 (4)	
Insured through employer				.001
Yes	208 (57)	84 (48)	117 (65)	
No	158 (43)	91 (52)	62 (35)	
Covered by spouse's or partner's insurance				.009
Yes	44 (12)	13 (7)	30 (17)	
No	322 (88)	162 (93)	149 (83)	
Has Apple Health, Medicaid, or similar government-assisted insurance				.006
Yes	39 (11)	27 (15)	11 (6)	
No	327 (89)	148 (85)	168 (94)	
Has Medicare				NA
Yes	4 (1)	2 (1)	2 (1)	
No	362 (99)	173 (99)	177 (99)	
Covered by Department of Veterans Affairs				NA
Yes	0			
No	366 (100)	175 (100)	179 (100)	
Purchased insurance directly from company				.026
Yes	17 (5)	13 (7)	4 (2)	
No	349 (95)	162 (93)	175 (98)	
Covered by Indian Health Service				NA
Yes	0			
No	366 (100)	175 (100)	179 (100)	
Has other insurance				.068
Yes	16 (4)	11 (6)	4 (2)	
No	350 (96)	164 (94)	175 (98)	
**Any food assistance[b], N (%)**				< .001
Yes	84 (23)	58 (33)	24 (13)	
No	282 (77)	117 (67)	155 (87)	
**Years worked in early education, mean (SD)**	10.10 (9.51)	5.99 (6.46)	13.82 (10.15)	< .001
**Professional society membership[c], N (%)**				.003
Yes	64 (19)	18 (11)	41 (24)	
No	277 (76)	141 (81)	130 (73)	
**Job title, N (%)**				< .001
Center Director	29 (8)	4 (2)	22 (12)	
Program Coordinator	17 (5)	1 (1)	16 (9)	
Lead Teacher	122 (33)	44 (25)	73 (41)	
Teacher	95 (26)	54 (31)	39 (22)	
Assistant Teacher	78 (21)	63 (36)	13 (7)	
Other (including 2 aides)	25 (7)	9 (5)	16 (9)	

SOURCE: Study Survey (ECE worker survey)

NOTES: NA is not applicable because there were too few respondents in some cells to reliably compute statistics.

Twelve records without wage data are not included in the wage columns. Percentages calculated without missing data. Chi square used for categorical variables, *t*-tests used for continuous variables, and median regression for comparison of medians for computation of p-values.

[a]This represents the total combined household income of all members in the household (including the ECE worker) over the past 12 months. This variable includes money from jobs, net income from business, farm or rent, pensions, dividends, interest, social security payments and any other monetary income received by members of the household whom were 15 years of age or older.

[b]Includes Supplemental Nutrition Assistance Program (n=43); Women, Infants, and Children (WIC) (n=27); Farmers market WIC program (n=6); food bank (n=15); reduced and free school lunch (n=32); other (n=1). Individuals participating in more than one program were only counted once.

[c]Professional Association such as the National Association for the Education of Young Children, the National Association for Family Child Care, the National Institute on Out of School Time, or a similar organization.

Appendix Exhibit A4. ECE Worker Health by Hourly Wage, further detail (n=366)

	All subjects	< site median hourly wage	≥ site median hourly wage	p-value
**Mental Well-being**				
**Depression CESD-R score[a],** mean (SD)	15.79 (10.94)	16.78 (11.93)	14.97 (9.98)	.125
**Categorical depression based on CESD-R score[a]:**				.084
Major, Probable, Possible, N (%)	26 (7)	18 (11)	8 (5)	
Subthreshold, N (%)	120 (33)	57 (34)	58 (33)	
No clinical significance, N (%)	213 (59)	95 (56)	111 (63)	
**Perceived stress score[b],** mean (SD)	23.6 (7.7)	24.7 (8.0)	22.8 (7.3)	.022
**Categorical stress based on perceived stress score:[c]**				.082
Low Stress < 20, N (%)	118 (33)	46 (27)	68 (38)	
Moderate Stress 20-36, N (%)	217 (60)	110 (65)	99 (56)	
High Stress >36, N (%)	25 (7)	14 (8)	11 (6)	
**SF-12[c] Mental Health Subscales,** mean (SD)				
Vitality	48.69 (9.46)	48.96 (10.05)	48.43 (8.73)	.594
Social functioning	44.66 (11.16)	42.95 (11.43)	46.19 (10.81)	.007
Mental health	46.56 (10.67)	46.25 (11.68)	46.74 (9.77)	.670
**Food Security**				
**Food security score[d],** mean (SD)	1.83 (2.20)	2.23 (2.29)	1.44 (2.03)	.001
**Categorical food security score:[d]**				.002
High or marginal 0-1, N (%)	212 (59)	86 (51)	119 (68)	
Low 2-4, N (%)	75 (21)	39 (23)	34 (19)	
Very low 5-6, N (%)	71 (20)	45 (26)	23 (13)	
**Physical Well-being**				
**Chronic Disease**, N (%)<br>Told by a doctor they have…				
**High blood pressure**				.240
Yes	62 (17)	26 (15)	35 (20)	
No	304 (83)	149 (85)	144 (80)	
**High cholesterol**				.032
Yes	54 (15)	19 (11)	34 (19)	
No	312 (85)	156 (89)	145 (81)	
**Diabetes**				.230
Yes	25 (7)	9 (5)	15 (8)	
No	341 (93)	166 (95)	164 (92)	
**Body Mass Index (BMI) (kg/m^2),** mean (SD)	29.08 (8.51)	28.74 (7.18)	30.06 (9.67)	.042
**Categorical BMI:**				.754
Underweight <18.5, N (%)	6 (2)	4 (2)	2 (1)	
Normal 18.5-24.9, N (%)	133 (38)	65 (39)	65 (37)	
Over weight 25-29.9, N (%)	96 (27)	45 (27)	46 (26)	
Obese 30+, N (%)	119 (34)	53 (32)	62 (35)	
**SF-12[c] Physical Health Subscales,** mean (SD)				
Physical functioning	50.26 (9.39)	50.37 (9.15)	50.23 (9.71)	.891
Bodily Pain	46.94 (9.86)	47.19 (9.58)	47.20 (9.63)	.999
General health	43.96 (11.65)	42.51 (11.59)	45.15 (11.67)	.034
**Health Behaviors**				
**Dietary Intake[e]**<br>**Number of times eaten per day,** mean (SD)				
Fruit and vegetable consumption	2.33 (1.34)	2.13 (1.21)	2.57 (1.44)	.002
Dairy consumption	0.92 (0.76)	0.92 (0.86)	0.94 (0.67)	.776
Sweets consumption	1.22 (1.14)	1.32 (1.18)	1.13 (1.11)	.128
**Physical Activity**<br>**IPAQ MET-min/week[f],** mean (SD)	3477 (3929)	3731 (4022)	3301 (3854)	.310

**Categorical physical activity based on MET minutes per week:[f]**				.294
Low Activity, N (%)	51 (14)	22 (13)	25 (14)	
Moderate Activity, N (%)	171 (48)	75 (44)	91 (51)	
High Activity, N (%)	138 (38)	73 (43)	62 (35)	
**Sleep duration (hours/night)[g],** mean (SD)	6.50 (1.13)	6.60 (1.17)	6.41 (1.08)	.122
**Categorical sleep per night:[g]**				.419
7 or more hours per night, N (%)	180 (49)	90 (51)	85 (47)	
<7 hours per night, N (%)	179 (49)	81 (46)	91 (51)	
**Tobacco use in last 30 days[h],** N (%)				.826
Yes	47 (13)	24 (14)	23 (13)	
No	316 (86)	150 (86)	154 (86)	
**Smoke e-cigarettes in last 30 days[h],** N (%)				.599
Yes	20 (5)	11 (6)	9 (5)	
No	345 (94)	163 (93)	170 (95)	

SOURCE: Data from the ECE worker survey.

Twelve records without wage data are not included in the wage columns. Percentages calculated without missing data. Chi square used for categorical variables and t-test used for continuous variables for computation of p-values. See Appendix for more detailed descriptions of measures and percentages.

[a]Center for Epidemiologic Studies Depression Scale-Revised (CESD-R) Depression Score is determined by responses to 20 questions and ranges from 0-60, with lower scores being fewer depressive symptoms. Scores allow for categorization into major, probable, possible, and subthreshold depression (scores <16 are categorized as "no clinical significance"). Subthreshold are scores above 16 but do not have two or more Diagnostic and Statistical Manual, 5th edition symptom groups occurring either nearly every day for the past two weeks, or 5-7 days in the past week and are considered clinically relevant because the probability of clinically validated diagnosis is high.

[b]Perceived stress scores determined by responses to 14 questions on the Perceived Stress Scale (PSS-14) and range from 0 to 56, with higher scores indicating greater perceived stress and can be categorized into low stress: <20; moderate stress: 20–36; high stress: >36.

[c]Short Form Health Survey (SF-12) consists of 12 items. Subscales range from 0-100, with higher scores representing above average health status, and are designed to have a mean score of 50 with a standard deviation of 10 in a representative sample of the U.S. population.

[d]Food Security Score is determined using the 6-item U.S. Household Food Security Survey Module. Raw scores range from 0 to 6, with high scores indicating very low food security.

[e]Dietary Screener Questionnaire (DSQ) asked "During the past month, how often did you eat…" for a number of food items. We grouped questions into food groups and calculated the number of times per day foods from a group were eaten

[f]International Physical Activity Questionnaire (IPAQ) long version asks 27 questions about 5 activity domains. The instrument produces outcome measures in minutes of weekly energy expenditures by intensity (MET-minutes/week).

[g]Number or usual hours of sleep per night.

[h]Using NHIS questions, participants were asked how often they smoked or used e-cigarettes in the last 30 days.

Appendix Exhibit A5. Distribution of ECE Workers by their Center Characteristics, by Hourly Wage (n=366)

	All Subjects N (%)	< site median hourly wage N (%)	≥ site hourly wage N (%)	p-value
**Number of children at center**				.028
50 or less	105 (30)	56 (33)	43 (26)	
51-75	104 (30)	58 (34)	44 (27)	
More than 75	138 (40)	56 (33)	78 (47)	
**Number of employees at center**				.012
14 or less	101 (29)	52 (31)	43 (26)	
15-30	167 (49)	90 (54)	75 (45)	
More than 30	76 (22)	25 (15)	47 (28)	
**Average hourly wage of full time employees at center**				< .001
$7.00-$13.00	79 (22)	56 (33)	19 (11)	
$13.01-$15.00	127 (35)	58 (34)	65 (37)	
$15.01-$21.00	154 (43)	57 (33)	94 (53)	
**Monthly enrollment fee for 4 yr olds at center**				.003
$750 or less	44 (13)	28 (17)	11 (6)	
$751-$1250	167 (48)	82 (50)	80 (47)	
$1251-2100	135 (39)	55 (33)	80 (47)	
**Profit status of center**				.946
Non-profit	165 (47)	77 (46)	83 (48)	
For-profit	148 (42)	73 (44)	73 (42)	
Community college or university affiliated	38 (11)	16 (10)	17 (10)	
**Center Participates in CACFP[a]**				< .001
Yes	155 (49)	93 (60)	52 (34)	
No	164 (51)	63 (40)	100 (66)	
**Center Participates in State QRIS[b]**				.040
Yes	286 (78)	144 (82)	131 (73)	
No	80 (22)	31 (18)	48 (27)	
**Center is NAEYC[c] accredited**				.463
Yes	91 (25)	41 (23)	48 (27)	
No	275 (75)	134 (77)	131 (73)	
**Center accepts state-level subsidy[d]**				.074
Yes	309 (84)	153 (87)	144 (80)	
No	57 (16)	22 (13)	35 (20)	
Center has at least one child currently enrolled on state-level subsidy program				.017
Yes	258 (70)	134 (77)	112 (63)	
No	108 (30)	41 (23)	67 (37)	
Center has more than 25% of enrolled children on state-level subsidy program				< .001
Yes	94 (40)	64 (50)	26 (27)	
No	143 (60)	64 (50)	71 (73)	
**Center accepts city-level subsidy programs[e]**				.598
Yes	183 (50)	88 (50)	85 (47)	
No	183 (50)	87 (50)	94 (53)	
Has at least one child currently enrolled on city-level subsidy program				.804
Yes	112 (41)	51 (40)	51 (38)	
No	160 (59)	77 (60)	82 (62)	
**Center offers health insurance**				< .001
Yes	252 (71)	110 (63)	134 (81)	
No	101 (29)	65 (37)	32 (19)	
**Center offers paid sick leave**				< .001
Yes	321 (88)	141 (81)	170 (95)	

No	45 (12)	34 (19)	9 (5)	
**Center offers parental/family leave**				< .001
Yes	281 (82)	116 (70)	156 (96)	
No	60 (18)	50 (30)	7 (4)	
Among those offering leave:				.100
Offers unpaid leave	198 (70)	88 (76)	104 (67)	
Offers paid leave	83 (29)	28 (24)	52 (33)	
**Center minimum education requirement for teachers**				.621
High school diploma/GED	278 (76)	132 (75)	139 (78)	
More than high school diploma/GED	88 (24)	43 (25)	40 (22)	

SOURCE: Data from the Center Director Questionnaire and attributed to workers from those centers.

Twelve records without wage data are not included in the wage columns. Chi square used for categorical variables for computation of p-values.

[a]Child and Adult Care Food Program
[b]Quality Rating and Improvement System
[c]National Association for the Education of Young Children
[d]This includes the Washington State Department of Social and Health Services (DSHS) and the Texas Child Care Management Services (CCMS) state subsidy programs for child care
[e]City programs include City of Seattle Child Care Assistance Program and Texas Workforce Solutions Capital Area Child Care Services

Appendix Exhibit A6. Key Themes and Illustrative Quotes from Focus Groups with Teachers and Center Directors (n=26)

**Theme 1. Emotional and mental health of teachers was of paramount importance.**
…when you're a giver, like you have a bucket and you give and you give…even when your bucket's empty. And at the end of the day, you have nothing. And I think that's what we do. We just give all day and we give everything we have. And at the end of the day sometimes we don't have anything for ourselves. –Teacher, SKC, WA
It's definitely a job that requires a lot of resilience. Yeah, it's just not a job that you can take lightly. –Teacher, Seattle, WA
Having support from my coworkers and director is key to dealing with that (stress). But also, leaving work at work. Go do yoga, get a massage, call my best friend, go outside, and know that I have to consciously do this so that I can get up in the morning at 5 a.m. and make it to work on time. –Teacher, Austin, TX
So sometimes when you're working with teachers it's looking at not only are they healthy enough to be the body in the classroom, physically, but are they mentally in a place where they're going to be able to actually engage with the children? And go back to that thriving thing where the teacher and the child are thriving. And I haven't found a magic key to make those things balance yet. –Director in SKC, WA
…a lot of people who are attracted to this profession have grown up in very traumatic circumstances themselves as children and so they have that inner desire to see that those things don't happen to other children coming up. –Director, Seattle, WA
The expectations for what teachers are supposed to be doing now have risen this much, and the pay has gone up about that much (makes narrow space between fingers). There's a constant feeling of 'I'm not doing enough.' –Director, Austin, TX
**Theme 2. Societal and parental disrespect were a major source of stress for ECE teachers**.
I give the same care to them (children in her care) that I would give to my own children, but they (the parents) don't see it that way. – Teacher in Austin, TX
You do it for the kids, but you also hope that the parents appreciate your work too. And when they don't it's like, that is depressing, that you worked so hard and loved their child all day and they don't appreciate you. –Teacher, SKC, WA
Is there a more important job? [Laughter] Like is there a more important job? I'm teaching your child to be a good human – Teacher, Seattle, WA
The bottom line is, in this field, compared to a lot of other fields, you can get personally attacked. Parents can be vicious. And I understand to an extent because they're doing that mama bear thing. But they become illogical. And they don't have any problem smearing a teacher or a childcare center all over social media. –Director, SKC, WA
I really think we're looking at a group of people (ECE teachers) who are so devalued in what they do that is has to have an impact on them. Even those who are maybe getting paid a little bit better. Families don't necessarily respect or value them. Our society doesn't. Our institutions don't. I worry about that. –Director, Austin, TX
**Theme 3. Directors wanted to support teachers' self-care, but faced staffing and other resource constraints.**
Who am I going to call in sick to? I have a very strong sense of obligation, but that's just the way I am. So I'm going to work and if I am that sick then I will hope that somebody will come in and then let me go. Or I'll divide my class up. We don't really have any … we don't have support staff at our work. Like we have a director and she's halftime director and halftime teacher. And so there's nobody, so it is hard because I feel guilty. –Teacher in SKC, WA
I would say my center, although there is the culture of promoting health, I mean we often have like chronic understaffing problems and turnover… I think they have the best intentions and maybe they do want to provide these things for us, but like pragmatically, it just … it can't happen. –Teacher, Seattle, WA
It's just such an in-your-face job. You have to be so responsive and so ready. The noise level and all of that kind of stuff. So if you're not emotionally stable and that comes at you, you have to take the teacher back and tell them to go take a walk and take a breather for 15 minutes... –Director, Austin, TX
I have a basket of different cards, I've got candy in my office, and I will … I might write them a note, I recognize you're having a rough day. –Director, SKC, WA
